# Conservative Management of Hemoperitoneum Due to a Ruptured Hemorrhagic Ovarian Cyst in Type 3 von Willebrand Disease

**DOI:** 10.7759/cureus.99168

**Published:** 2025-12-13

**Authors:** Hounaida Mahfoud, Rim Laaboudi, Mohamed Elkhorassani, Nabil Elachhab, Samir Bargach

**Affiliations:** 1 Obstetrics and Gynecology, Ibn Sina University Hospital, Rabat, MAR; 2 Obstetrical Gynecology and High-Risk Pregnancy, Maternity Souissi, Rabat, MAR; 3 Pediatrics Hematology and Oncology, Ibn Sina University Hospital, Rabat, MAR; 4 Maternal Critical Care and Anesthesiology, Ibn Sina University Hospital, Rabat, MAR

**Keywords:** conservative management, hemoperitoneum, ruptured hemorrhagic cyst, von willebrand disease, von willebrand factor replacement

## Abstract

Hemoperitoneum secondary to a ruptured hemorrhagic ovarian cyst is usually a benign and self-limiting condition, but in women with type 3 von Willebrand disease (VWD), it may evolve into a severe and potentially life-threatening event due to the inability to achieve spontaneous hemostasis. We report a case of a 30-year-old woman with type 3 VWD who presented with acute pelvic pain and profound anemia. Transvaginal ultrasonography and MRI demonstrated a ruptured hemorrhagic ovarian cyst associated with a large hemoperitoneum. Despite the extent of bleeding, the patient remained hemodynamically stable and was managed conservatively. Treatment consisted of von Willebrand factor replacement, blood transfusion, analgesia, and close clinical and radiological monitoring. Her condition improved progressively, with normalization of hemoglobin levels, resolution of symptoms, and significant regression of the hemoperitoneum on follow-up imaging. No surgical intervention was required. Managing ovarian cyst rupture in severe VMD is particularly challenging, as surgical intervention carries substantial hemorrhagic risk. Conservative management, when supported by adequate hemostatic therapy, can be both safe and effective, preserving ovarian function and reducing perioperative morbidity. This case underscores the importance of individualized, multidisciplinary care to optimize management strategies in women with severe bleeding disorders.

## Introduction

Von Willebrand disease (VWD) is the most common inherited bleeding disorder and represents a major cause of gynecological hemorrhage in women, most frequently manifesting as menorrhagia. However, VWD may also lead to less common but potentially serious complications such as hemoperitoneum secondary to the rupture of a hemorrhagic ovarian cyst, an event that is typically benign and self-limiting in women without coagulopathies. In patients with VWD, the absence or deficiency of von Willebrand factor prevents adequate spontaneous hemostasis, allowing continued bleeding after cyst rupture and resulting in significant hemoperitoneum that may lead to hemodynamic instability. Although surgery is the conventional management in cases of acute intraperitoneal bleeding, operative treatment carries an additional hemorrhagic risk in VWD, particularly in severe forms such as type 3. For this reason, conservative management supported by appropriate hematologic therapy is often the preferred therapeutic strategy [1-6].

We hereby present a case of hemoperitoneum secondary to a ruptured hemorrhagic cyst in a patient with type 3 VWD, successfully managed using a conservative approach.

## Case presentation

A 30-year-old woman, gravida 0 para 0, with a known history of type 3 VMD diagnosed at the age of two, presented to the gynecological emergency department. Throughout childhood and adolescence, she experienced recurrent episodes of epistaxis, gingivorrhagia, and menorrhagia, requiring multiple blood transfusions and repeated administrations of von Willebrand factor. She had been undergoing ovulation-induction therapy for the past three months for primary infertility lasting five years.

The patient reported acute pelvic pain evolving over five days, progressively worsening, and associated with asthenia. She denied any menorrhagia or history of abdominal trauma.

On examination, she was tachycardic at 112 bpm but normotensive at 110/60 mmHg. She appeared pale but remained apyretic. Abdominal palpation showed diffuse tenderness without guarding. Gynecological examination revealed a normal cervix with no active bleeding. Vaginal examination identified a left adnexal, smooth, mobile, and tender mass and elicited a marked Douglas sign.

**Figure 1:**
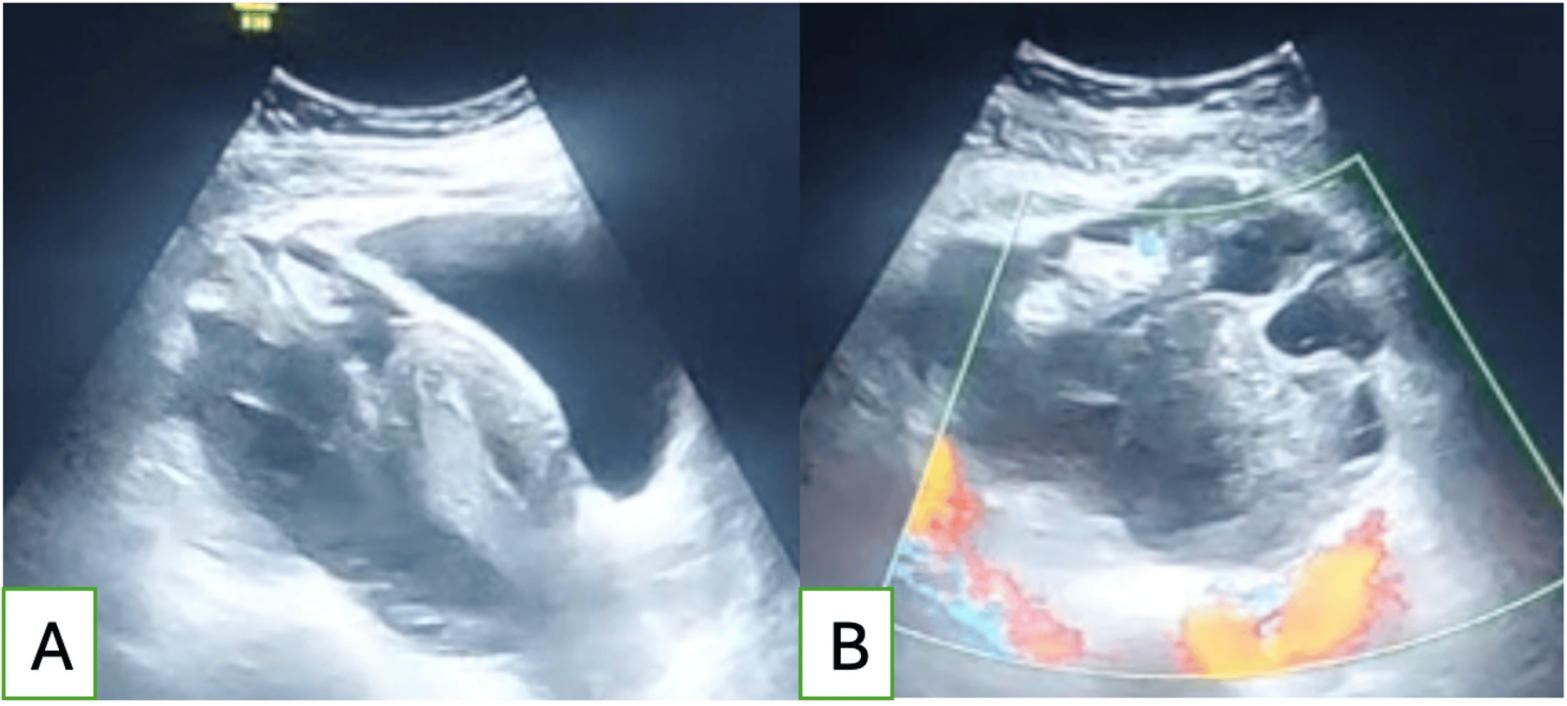
Ultrasound images of a left adnexal mass suggestive of a ruptured hemorrhagic cyst (A) Sagittal view showing the uterus with a hemorrhagic cyst. (B) Axial view of the cyst with color Doppler.

Transvaginal ultrasound demonstrated a left adnexal mass measuring 65 × 46 mm, suggestive of a ruptured hemorrhagic ovarian cyst, as well as a large-volume anechoic peritoneal effusion extending into the hepatorenal and splenorenal recesses, the paracolic gutters, and the pelvis, consistent with hemoperitoneum (Figure 1). 

Laboratory tests showed a low hemoglobin of 3.5 g/dL, normal inflammatory markers (WBC, CRP), and negative beta-human chorionic gonadotropin (β-hCG). Hemostasis parameters were normal, notably a platelet count of 266,000/µL, prothrombin activity of 72%, and international normalized ratio (INR) of 1.19. However, the von Willebrand factor level was severely decreased to <0.1%.

The patient was admitted to the ICU and received four units of packed red blood cells and Wilate at 30 mg/kg every 12 hours for 48 hours. Antibiotic prophylaxis was initiated to prevent secondary infection of the hemoperitoneum. The patient was closely monitored and showed significant improvement in pelvic pain and general state, with hemoglobin levels rising to 7 g/dL after transfusion.

**Figure 2:**
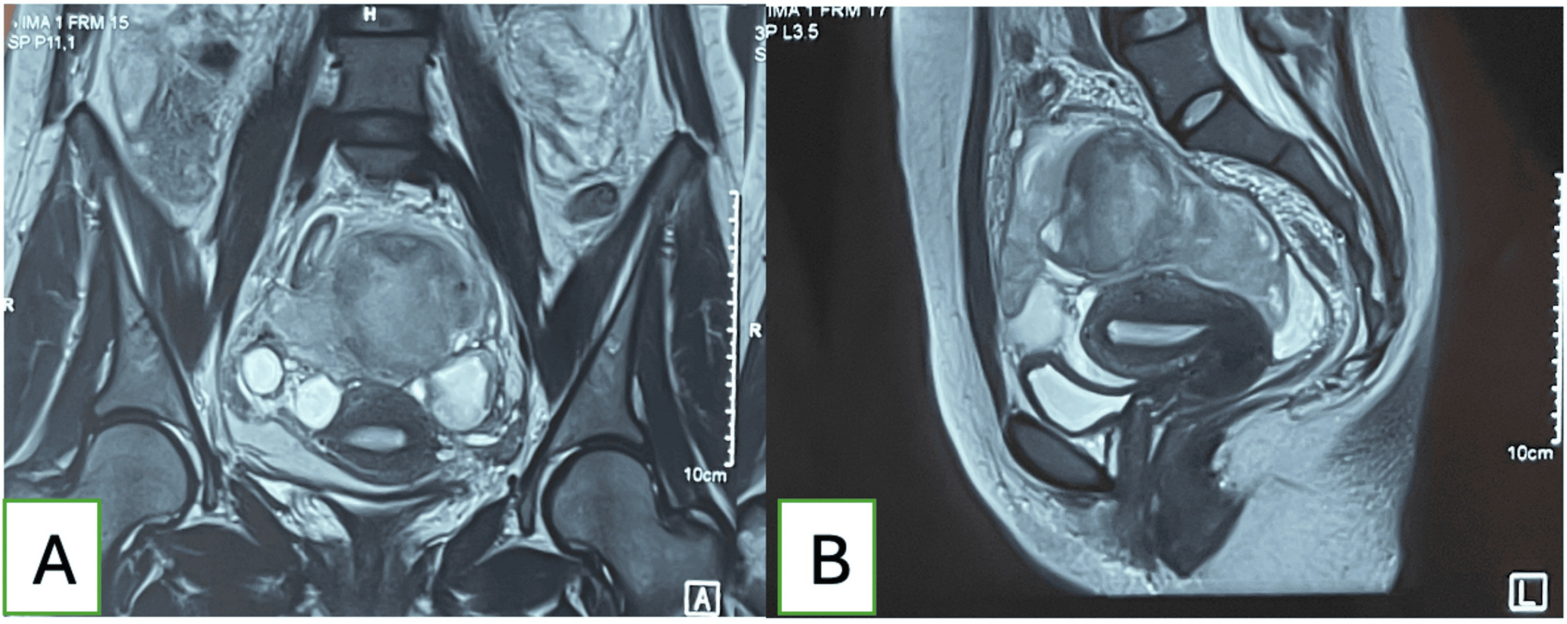
MRI images showing moderate hematic pelvic effusion with a left hemorrhagic ovarian cyst (A) Axial view showing a left hemorrhagic cyst and right hydrosalpinx. (B) Sagittal view showing moderate hematic pelvic effusion and a suprauterine hematoma.

An MRI to better assess the hemoperitoneum was performed the following day and revealed a suprauterine hematoma measuring 53 × 57 × 57 mm, a moderate hematic pelvic effusion more pronounced in the vesicouterine space, a left hemorrhagic ovarian cyst measuring 30 × 41 mm, and a right hydrosalpinx (Figure 2). 

Given her hemodynamic stability, improvement in pain, and radiological stability of the hemoperitoneum, a conservative management strategy was adopted.

On day 3, she received two additional units of blood, leading to a hemoglobin increase to 11 g/dL, and her von Willebrand factor level rose to 136% after replacement therapy. A follow-up ultrasound one week later showed a marked regression of hemoperitoneum, while the hemorrhagic cyst remained stable in size.

The patient was discharged on continuous combined oral contraceptives to suppress ovulation and reduce the risk of recurrent hemorrhagic ovarian cysts, along with weekly Wilate infusions (30 mg/kg) and iron supplementation. She was also scheduled for weekly clinical assessments and transvaginal ultrasound monitoring. 

At six weeks post-event, she remained asymptomatic, with stable hemoglobin levels above 10 g/dL. Further counseling is planned to evaluate her ongoing infertility concerns.

## Discussion

Hemoperitoneum of gynecological origin may arise from potentially life-threatening conditions such as ectopic pregnancy, ovarian torsion, or uterine rupture. However, it can also result from benign etiologies, including retrograde menstrual flow, endometriosis, or ovulation [1]. In fact, during the periovulatory period, rupture of a follicular or functional ovarian cyst may occur, leading to intracystic hemorrhage. When the intracystic pressure increases, the cyst may rupture into the peritoneal cavity, generating a hemoperitoneum of variable severity [2].

Clinically, the presentation ranges widely: minimal hemoperitoneum may manifest as mild pelvic cramps, while more significant bleeding can cause abdominal tenderness, peritoneal signs like nausea and vomiting, and even hemodynamic instability. On pelvic examination, a lateral adnexal mass may be palpated; more frequently, a positive Douglas sign is found [3].

Diagnosis is primarily supported by transvaginal ultrasonography, which typically reveals a round ovarian lesion of variable size depending on whether the cyst is follicular or functional. Ultrasonography also characteristically detects hemoperitoneum, particularly in dependent spaces such as the pouch of Douglas, the vesicouterine pouch, the iliac fossae, and, when abundant, the Morrison pouch and paracolic gutters. The volume of intraperitoneal blood may range from minimal to massive [4]. MRI is a valuable complementary exam, allowing better characterization of ovarian lesions by distinguishing benign from malignant features and providing a more accurate assessment of the volume, distribution, and hematic nature of the peritoneal effusion [5].

Management relies mainly on two therapeutic strategies: conservative (wait-and-see) or surgical. Conservative management requires close monitoring, analgesia, antibiotic prophylaxis, and, when needed, antifibrinolytic agents. Blood transfusion may be necessary in cases of significant anemia. Conversely, in patients showing hemodynamic instability or rapid expansion of intraperitoneal bleeding, urgent laparoscopy is mandatory to control the source of bleeding, either by cystectomy or oophorectomy [2-4].

Type 3 VWD is the rarest and most severe form, characterized by an almost complete absence of von Willebrand factor. Von Willebrand factor plays a crucial role in primary hemostasis by mediating platelet adhesion and stabilizing factor VIII. Its severe deficiency, therefore, leads to profound bleeding tendencies [6].

The most frequent gynecological bleeding in patients with VWD is menorrhagia, with reported prevalence ranging from 32% to 100% [6]. It is therefore recommended to investigate underlying bleeding disorders in adolescents and young women presenting with heavy menstrual bleeding, as this may be the first clinical manifestation of VWD [7]. Diagnosis relies on measurement of von Willebrand factor antigen and activity, which are typically undetectable in type 3 disease [6].

Management of gynecological bleeding in VWD is based on ovulation suppression by continuous combined oral contraception, regular von Willebrand factor replacement therapy, and correction of iron deficiency anemia [4]. Despite hormonal control, women with VWD remain prone to additional gynecological bleeding events, among them hemorrhagic ovarian cysts, the second most common cause of bleeding in this population, with reported incidence between 6.8% and 52% [6-8]. Hemorrhage may occur during ovulation due to bleeding from the ruptured follicle and can be precipitated by coitus, trauma, intense physical activity, or even pelvic examination. In VWD, these cysts may continue to bleed due to the absence of adequate coagulation factors, making management challenging [4]. 

The literature highlights several essential principles regarding the management of hemoperitoneum in patients with VMD. Most authors agree that conservative management should be favored whenever safely possible. Surgery is generally considered a last resort because VWD patients carry a significantly increased bleeding risk, hemorrhagic cysts tend to recur, and repeated ovarian surgery may compromise future fertility [9]. Successful conservative treatment relies on aggressive hemostatic replacement therapy, such as Wilate or Humate-P, blood transfusions when necessary, and close clinical and ultrasound monitoring. In type 3 VWD, desmopressin is ineffective, which further underscores the importance of von Willebrand factor concentrates in the acute setting [1-10].

Surgical intervention is reserved for well-defined situations, particularly when hemodynamic instability persists despite optimal hemostatic therapy, when imaging confirms ongoing active bleeding, or when another acute surgical condition cannot be excluded, such as adnexal torsion or rupture of a neoplasm. In such cases, the literature emphasizes the need for preoperative correction of von Willebrand factor levels, coordinated management with hematology, and the use of minimally invasive techniques whenever feasible [3].

Prevention also plays a crucial role in the long-term management of these patients. Given the high rate of recurrence of hemorrhagic ovarian cysts in VWD, the authors recommend long-term ovulation suppression through continuous combined oral contraception, cyclic inhibition, or, in selected cases, gonadotropin-releasing hormone (GnRH) analogues. Scheduled von Willebrand factor replacement therapy is particularly beneficial in severe type 3 disease. Patient education is equally important, particularly regarding the avoidance of high-impact physical activities during periovulatory phases, which may precipitate cyst rupture [4].

Finally, fertility preservation remains a central consideration. Conservative management helps maintain ovarian reserve and prevents the cumulative harm associated with repeated adnexal surgeries. This is especially relevant for women undergoing infertility treatment, as in the present case. Overall, the literature supports a strategy prioritizing fertility preservation, long-term hormonal regulation, and meticulous hematologic optimization [9].

## Conclusions

Hemoperitoneum from a ruptured hemorrhagic ovarian cyst in type 3 VMD presents a complex therapeutic challenge as surgical intervention carries substantial bleeding risk, while conservative management is often favored. This case illustrates that a carefully monitored non-surgical approach, supported by appropriate hematologic therapy, can be both safe and effective. Nevertheless, it remains unclear whether the repeated spontaneous resolution of large hemoperitoneums may predispose to peritoneal adhesions and potentially affect future fertility. Further studies are needed to clarify these long-term implications and to guide optimal management strategies in women with severe bleeding disorders.
